# 572 Burn injury from smoking electronic cigarettes while on supplemental oxygen

**DOI:** 10.1093/jbcr/irac012.200

**Published:** 2022-03-23

**Authors:** Steven A Kahn, Kathleen Hollowed, Deepak Ozhathil

**Affiliations:** Medical University of South Carolina, Charleston, South Carolina; South Carolina Burn Center at MUSC, Charleston, South Carolina; Medical University of South Carolina, Charleston, South Carolina

## Abstract

**Introduction:**

Burn injuries from exploding electronic cigarettes have been well documented in the medical literature and lay press, as have injuries from smoking conventional cigarettes while on supplemental oxygen. However, there is a paucity of literature regarding injuries from smoking electronic cigarettes while on supplemental oxygen. Empirically, it might seem safe to use e-cigarettes while on oxygen, but it can result in explosion. The electronic cigarette coil can heat up to 350°C and serve as a source of ignition. The purpose of this study is to describe and characterize this relatively novel and uncommon mechanism of injury.

**Methods:**

This study was a descriptive review of 2013-2016 National Burn Repository (NBR) Data. Injury description fields were queried for “oxygen”, “O2”, “electronic cigarettes”, and various abbreviations and misspellings. Demographics, burn size, length of stay, expected length of stay using 1.1 days per %TBSA, and LOS index were reported. In addition, a Google search for lay press articles using the terms “smoking,” “’oxygen,” and “electronic (or e) cigarettes was performed, and these cases were tabulated.

**Results:**

Of approximately 60,000 NBR entries, only 8 records of injury while smoking e-cigs on oxygen were found. Patients were predominantly male (63%) with a mean age of 63±9 years old, burn size of 3.4%±4 TBSA and LOS of 5.8±7 days. Inpatient stay averaged 1.65 days per %TBSA, which was 150% of expected LOS for the cohort. Two patients sustained third degree burns of 0.5% and 11%TBSA. Three patients were intubated, and had a mean of 3.33 ventilator days. Most injuries occurred at home (88%), while one occurred in a hospital. All 8 patients had Medicare and none of them suffered mortality. A google search revealed three distinct lay press articles regarding this topic.

**Conclusions:**

Although an uncommon mechanism of injury, smoking electronic cigarettes on supplemental oxygen can result in injury and a significant hospital stay. Limitations in abstraction from the database and/or reporting might have resulted in an under capture of the true number of injuries. Circumstances resulting in this phenomenon warrant further investigation, as it is unclear if it occurs from malfunction or a hot coil alone. Patients who are prescribed home O2 should be warned about this potential danger.

